# Quantitative assessment of baseline imbalances in evolocumab and alirocumab trials: a meta-epidemiological study

**DOI:** 10.1186/s12874-024-02260-z

**Published:** 2024-06-22

**Authors:** F. H. van Bruggen, S. U. Zuidema, H. J. Luijendijk

**Affiliations:** Department of General Practice and Elderly Care Medicine, University of Groningen, University Medical Centre Groningen (UMCG), PO Box 196, Groningen, AD 9700 The Netherlands

**Keywords:** Baseline imbalances, PCSK9 inhibitors, Meta-regression, Bias

## Abstract

**Background:**

Baseline imbalances have been identified in randomized trials of evolocumab and alirocumab. Our aim was to quantitatively assess (1) the presence of systematic baseline differences, and (2) the relationship of baseline differences with effects on low-density lipoprotein-cholesterol (LDL-c) and clinical outcomes in the trials.

**Methods:**

We performed a meta-epidemiological study. PubMed, Embase, regulatory reports, ClinicalTrials.gov and company websites were searched for trials. Seven baseline characteristics (mean age, LDL-c, BMI, percentage males, diabetics, smokers, and hypertensives) and five outcomes (LDL-c, major adverse cardiac events, serious adverse events, any adverse events, all-cause mortality) were extracted. We calculated (1) range and distribution of baseline imbalances (sign-test), (2) pooled baseline differences and heterogeneity (meta-analysis), (3) differences in SDs around continuous variables (sign-test and pooling), and (4) the relationship of baseline differences with outcomes (meta-regression). The comparisons of PCSK9-inhibitor groups with either placebo or ezetimibe were analysed separately and combined.

**Results:**

We identified 43 trials with 63,193 participants. Baseline characteristics were frequently missing. Many trials showed small baseline imbalances, but some large imbalances. Only baseline BMI showed a statistically significant lower pooled mean for the drug versus placebo groups (MD -0.16; 95% CI -0.24 to -0.09). Heterogeneity in baseline imbalances was present in six placebo- and five ezetimibe-comparisons. Heterogeneity was statistically significant for BMI, males, diabetics and hypertensives in the combined comparisons. There was a statistically significant preponderance for larger SDs in the PCSK9-inhibitor versus control groups (sign-test age 0.014; LDL-c 0.014; BMI 0.049). Meta-regression showed clinically relevant relationships of baseline imbalances in age, BMI and diabetics with the risk of any adverse events and the risk of mortality. Two relationships were statistically significant: A higher mean BMI in the drug versus control group with a decreased risk of mortality (beta − 0.56; 95% CI -1.10 to -0.02), and a higher proportion of diabetics with an increased risk of any adverse events (beta 0.02; 95% 0.01 to 0.04).

**Conclusions:**

Heterogeneous baseline imbalances and systematically different SDs were present in evolocumab and alirocumab trials, so study groups cannot be assumed to be comparable. These findings raise concerns about the design and conduct of the randomization procedures.

**Supplementary Information:**

The online version contains supplementary material available at 10.1186/s12874-024-02260-z.

## Background

Randomization is a core feature of a clinical trial. The goal of randomization is to balance known and unknown prognostic patient characteristics across the intervention and control group. If the groups are incomparable at baseline, a difference between the groups in outcomes at the end of the trial cannot be attributed to the intervention. Despite adequate randomization procedures, baseline imbalances may nevertheless occur due to chance especially in small trials. Also, inadequate design or application of randomization procedures may lead to systematic baseline imbalances [1, 2].

Baseline imbalances have been observed in trials of evolocumab and alirocumab [3]. These PCSK9 inhibitors were registered in 2015 when multiple lipid-lowering trials had shown that they reduce cholesterol effectively. Subsequently, clinical outcomes trials were performed to test whether the drugs decrease the risk of myocardial infarction, stroke, and death. The FOURIER trial tested evolocumab in 27,564 patients, and the ODYSSEY OUTCOMES trial tested alirocumab in 18,924 patients [4, 5].

A Cochrane review of these trials has shown small absolute effects of PCSK9 inhibitors on clinical outcomes. Alirocumab reduced the risk of cardiovascular disease compared to placebo (OR 0.87; 95% CI 0.80–0.94; RD -0.02; 95% CI -0.02 to -0.01), and the risk of all-cause mortality (OR 0.83; 95% CI 0.72–0.96; RD -0.01; 95% CI -0.01 to 0.00) [6]. Evolocumab also reduced the risk of cardiovascular disease compared to placebo (OR 0.84; 95% CI 0.78–0.91; RD -0.02; 95% CI -0.02 to -0.01) but not the risk of all-cause mortality risk (OR 1.04; 95% CI 0.91–1.19; RD 0.00; 95% CI -0.00 to 0.01). Neither drug affected the risk of cardiovascular disease or all-cause mortality risk more than ezetimibe. The clinical outcomes trials had a large weight in the meta-analyses of this Cochrane review.

However, the clinical outcomes trials of evolocumab and alirocumab have shown baseline imbalances. FOURIER reported a baseline difference in mean body weight (85.0 kg in the evolocumab group versus 85.5 kg in the placebo group) and in the percentage smokers (28.0% versus 28.5%) [4]. ODYSSEY OUTCOMES reported a baseline difference in the percentage of participants with hypertension (65.6% in the alirocumab group versus 63.9% in the placebo group) [5]. Smaller trials that tested the efficacy of PCSK9 inhibitors in terms of LDL-c lowering also reported baseline imbalances [7, 8]. Baseline differences should be adjusted for in the analyses of trials to avoid biased effects, but this is not commonly done [3, 9, 10].

Although baseline differences in trials are usually small, they may be relevant for several reasons. First, multiple small baseline differences in favor of the intervention group may together result in a biased overestimation of the treatment effect in the same direction, especially if there are few imbalances in favor of placebo. Secondly, pooling results from trials with comparable systematic baseline imbalances might lead to biased pooled effects [11–13]. Thirdly, an imbalance of a given absolute size will have a greater effect on the estimated effect in a large than a small study [14]. Fourthly, the presence of baseline imbalances raises the question whether the randomization procedure in the trial was adequately designed and executed.

Quantitative assessment of baseline imbalances might identify systematic baseline imbalances that could have affected the estimated treatment effect [13, 15]. Various methods for evaluating potential baseline imbalances in a set of trials have been documented. These methods involve meta-analysis of baseline differences, evaluation of heterogeneity in baseline differences, and meta-regression [2, 13, 15]. Especially age may be an important baseline variable in evaluating baseline imbalances [2]. The aim of this meta-epidemiological study was to assess the presence of systematic baseline imbalances in trials of evolocumab and alirocumab, and their association with the reduction of LDL-c and the risk of cardiovascular outcomes.

## Methods

We performed a meta-epidemiological study of randomized trials that compared evolocumab and alirocumab with placebo or ezetimibe among adult patients with hypercholesterolemia at an increased risk of cardiovascular disease. We reported this study following the guideline for reporting meta-epidemiological studies (see online supplement) [16].

### Search and selection

We used three sources to find phase 2 and phase 3 randomized trials of evolocumab and alirocumab. We searched the bibliographies PubMed and Embase with the terms ‘evolocumab, alirocumab and ‘placebo or ezetimibe’. Next, we checked the references of FDA reports, EMA reports, and a Cochrane review [17–22]. Cochrane reviews pay particular attention to identifying all available trials, also from the grey literature, to avoid publication bias. Finally, we looked for trials that were registered on ClinicalTrials.gov, and websites of the pharmaceutical companies manufacturing the before mentioned drugs. Our search was rerun for the last time in March 2023.

If a title and abstract suggested an eligible trial, we assessed the full text publication or protocol. We selected trials that were: (1) randomized, (2) placebo- and/or ezetimibe-controlled, and (3) performed among adult patients at an increased risk of cardiovascular disease. Phase 1, head-to-head, and open label (extension) trials were excluded. Language and publication date were not exclusion criteria. No study protocol was registered (see online supplement).

### Data extraction

Two independent reviewers (FvB and HJL) extracted the data with a standardized data form. First, we registered general study characteristics including investigated drug, number of participants, randomization procedure (method of random sequence generation and allocation concealment) and whether baseline imbalances had been adjusted for.

Secondly, we extracted the following baseline characteristics per treatment group: mean age (SD), percentage of males, mean LDL-c concentration (SD), body mass index (BMI) (SD), percentage with diabetes mellitus (DM), percentage of smokers, and percentage with hypertension. We chose these parameters because they are associated with the level of LDL-c serum concentration or the occurrence of cardiovascular disease, which were the primary outcomes of the lipid-lowering trials respectively clinical outcomes trials. When a standard deviation (SD) was missing, we used other reported data (e.g. the standard error) if available to calculate it [23].

Thirdly, we extracted various clinical outcomes per treatment group. We extracted change in LDL-c from baseline to the first measurement (usually at 12 weeks) after start of the treatment (in mg/l). When LDL-c change was reported as a percentage, we converted it to the absolute change. We also extracted the number of positively adjudicated major adverse cardiovascular events (MACE) as defined by the trial investigators. Finally, the number of any adverse events, serious adverse events (SAE) and all-cause deaths were extracted. SAE include all serious disease - both targeted and unintended - that is potentially fatal or causes permanent health damage [24, 25].

We included data from all randomized participants, except those in PCSK9 groups that differed from the comparison group in another way than just the allocated intervention. For instance, some trials used different doses of background statin therapy for the PCSK9 inhibitor and the comparison group. Intervention groups that received other active drugs than the PCSK9 inhibitors or ezetimibe were also excluded. If multiple dosages of the PCSK9 inhibitor were investigated in a trial, we combined the groups. The same applied to multiple placebo groups.

Our primary source of information for the baseline and outcome data were the published papers of the trials. If baseline data or outcomes data were not available in published papers, we used the data published at ClinicalTrials.gov. Disagreements about the data were resolved in consensus meetings.

### Statistical analysis

We performed three types of analyses to examine the presence of systematic baseline imbalances. First, we described the range and distribution of the baseline imbalances of the PCSK9 inhibitor groups (evolocumab or alirocumab) versus the comparison groups (placebo or ezetimibe). A baseline difference was present if the intervention group differed from the control group in terms of the figures (and number of decimals) presented by the authors. We run a one-sided sign-test for each baseline characteristic to find out if the direction of the baseline imbalance was more often positive or negative than could be expected by chance.

A sign-test takes into account the number of the comparisons and the direction of the baseline differences, but not the absolute size of the differences. The sign-test automatically excludes studies with no baseline imbalance and studies with missing baseline differences.

Secondly, we performed meta-analyses to calculate the pooled mean difference (MD) with a 95% CI for baseline age, LDL-c and BMI, and pooled risk difference (RD) for percentage males, percentage participants with DM, percentage smokers and percentage participants with hypertension between treatment groups. We used fixed effects models, because heterogeneity for pooled baseline differences should be 0% [2]. The analysis generated an I^2^-statistic for heterogeneity, and a p-value. We calculated 95% confidence intervals around I^2^ with the direct command heterogi in Stata. Trials with missing data were omitted from the analysis.

Thirdly, we described the range and distribution of the SDs around mean age, LDL-c and BMI of the PCSK9 inhibitor groups versus the comparison group. Again, we run a one-sided sign-test for the direction of imbalances. Next, we calculated pooled SDs with the standard formula to investigate whether SDs of the intervention and control groups differed systematically [26]. We performed a t-test for means between two groups to investigate systematic differences between pooled SDs. In addition, we used Levene’s test for differences between SDs in individual trials and describe the distribution of the generated p-values, which can be expected to vary evenly between 0 and 1.

We ran the abovementioned analyses for the placebo-controlled and ezetimibe-controlled trials separately, because we expected that systematic baseline differences might be different between those types of trials. This assumption was based on the findings of a study on baseline imbalances in placebo-controlled trial testing atypical antipsychotics in dementia. Some of these trials had an additional (third) haloperidol group, and these exhibited larger pooled imbalances than trials without this extra group [15]. If a trial had a placebo and an ezetimibe arm, the placebo arm was used in the analysis of placebo-comparisons and the ezetimibe arm in ezetimibe-comparisons. In addition, we rerun the analyses of the baseline differences for alirocumab and evolocumab trials separately, and we checked whether the pooled baseline differences and pooled SDs of the placebo-controlled trials might have been driven primarily by the (very) large clinical outcomes trials.

Finally, we performed univariate meta-regression analyses to evaluate the relationship of an individual baseline imbalance with the effects on clinical outcomes. We combined the data from all trials because the direction of the effect can be expected to be independent of type of control drug. We used random effects meta-regression as generally recommended [23]. As the SAE data of clinical outcomes trials were not reported according to MedDRA standards [27, 28], they were omitted from these meta-regression analyses. We performed a sensitivity analysis excluding the clinical outcomes trials at the request of a reviewer.

## Results

**Fig. 1:**
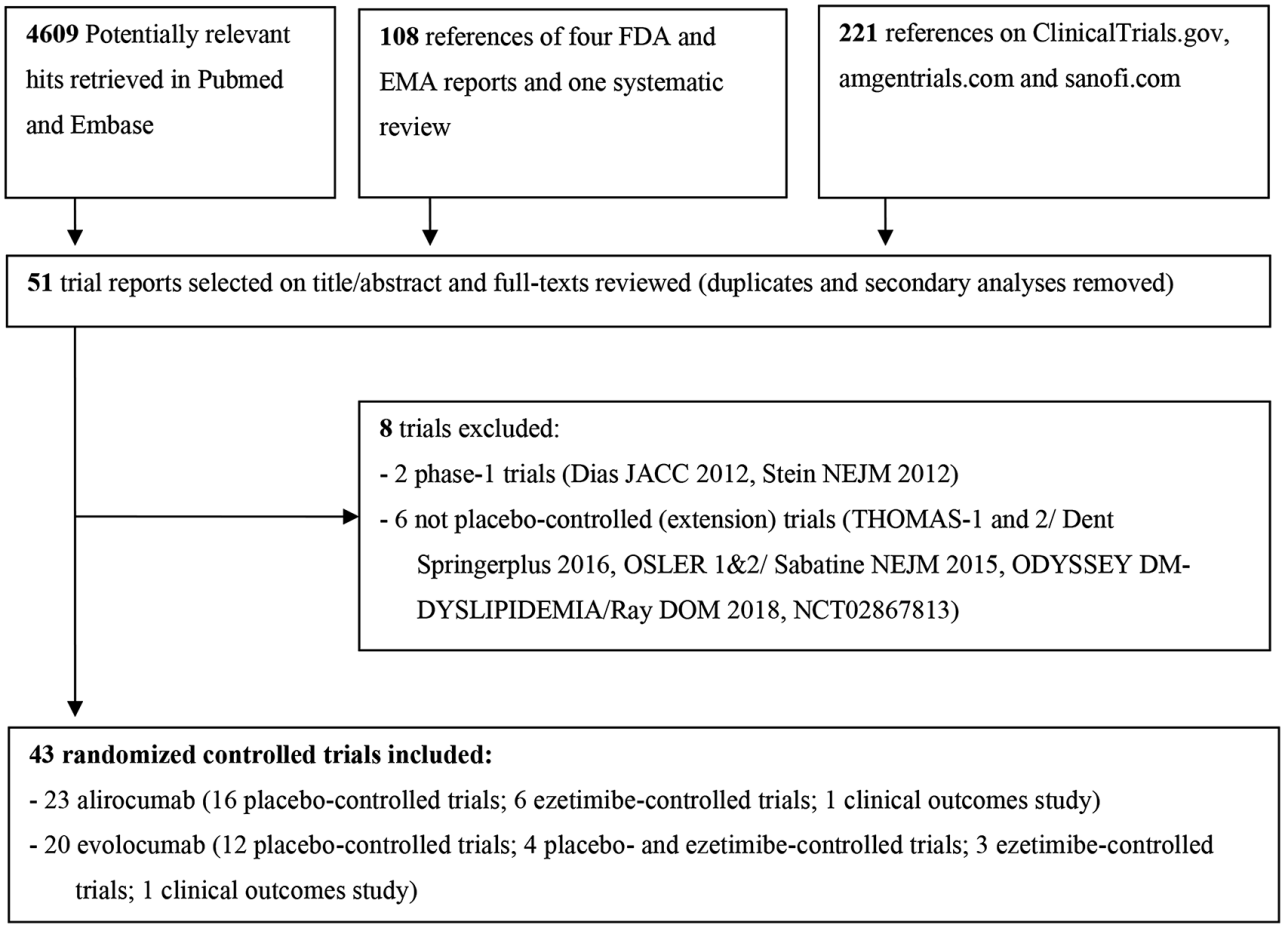
Flow diagram of literature search and study selection

Our search yielded 51 potentially eligible trials (Fig. 1). We found that all the planned trials reported in the FDA reviews have been published. After full-text assessments, we included 43 completed trials with 63,193 participants [4, 5, 7, 8, 28–66]. Among the included trials, there were 26 placebo-controlled trials, 13 ezetimibe-controlled trials, and 4 had a placebo and ezetimibe group (Table 1). The placebo-controlled trials included two clinical outcomes studies: FOURIER (evolocumab) and ODYSSEY OUTCOMES (alirocumab). Hence, the trials produced 30 placebo comparisons and 13 ezetimibe comparisons.

### Study characteristics

Twenty trials tested evolocumab in 36,050 patients and 23 alirocumab in 27,143 patients (Table 1). The majority of trials was conducted in patients with primary hypercholesterolemia, homozygous or heterozygous familiar hypercholesterolemia, or a combination. Most patients already used a statin, or were prescribed statins before randomization.

The methods for random sequence generation were reported in 5 of 43 trials, and for allocation concealment in 13 trials (Table 1). If described, the methods mainly concerned an interactive voice response system (IVRS) or interactive web response system (IWRS). Baseline imbalances were not adjusted for in any of the trials. Baseline age, sex and LDL-c was reported for all 30 placebo comparisons, but BMI for just a part of them (22/30), as was DM (24/30), smoking (18/30) and hypertension (17/30). The two clinical outcomes trials reported all seven baseline characteristics. Baseline age, sex, LDL-c and diabetes was reported for all 13 ezetimibe comparisons, but BMI (9/13 trials), smoking (7/13) and hypertension (10/13) for just a part of them.

### Range and distribution of baseline differences

Table 2 shows the range and distribution of the imbalances in the seven patient characteristics of interest. Most placebo comparisons showed small imbalances, but some large imbalances, as represented by large ranges for difference in percentage males (-19.6 to 25.8), mean LDL-c (-7.7 to 35.4), mean BMI (-1.5 to 1.5), percentage DM (-12.6 to 13.2), percentage smokers (-12.6 to 7.6) and percentage hypertension (-24.0 to 13.7). The ezetimibe comparisons showed small ranges in imbalances for mean age, mean LDL-c, and percentage smoking, but larger ranges for percentage males (-11.4 to 7.2), mean BMI (-0.7 to 1.7), percentage DM (-9.4 to 17.4), and percentage hypertension (-11.5 to 10.0).

Table 2 also presents the distribution of positive and negative baseline differences for the compared groups. Statistically significantly more trials had a higher percentage of men in the drug group compared to the placebo group (20 versus 9; *p* = 0.03). Although numerically more trials had a higher mean LDL-c in the drug group compared to the placebo group (18 vs. 10; *p* = 0.09), no other statistically significantly divergent distributions were found.

**Table 1:** Characteristics of included alirocumab and evolocumab trials

Study	Acronym	PCSK9 inhibitor	Sample size, n*	Random sequence generation	Allocation concealment	Adjustment for baseline imbalances	Missing baseline data	
*Lipid lowering placebo-controlled trials*								
McKenney 2012	DFI11565	alirocumab	183	NR	NR	No	none	
Roth 2012	DFI11566	alirocumab	61	NR	NR	No	none	
Stein 2012	CL-1003	alirocumab	77	NR	IVRS	No	smoking, HT	
Kastelein 2015	ODYSSEY FH I	alirocumab	486	NR	NR	No	none	
Kastelein 2015	ODYSSEY FH II	alirocumab	249	NR	NR	No	none	
Kereiakes 2015	ODYSSEY COMBO I	alirocumab	316	NR	NR	No	smoking, HT	
Robinson 2015	ODYSSEY LONG TERM	alirocumab	2338	CG	CAS	No	HT	
Ginsberg 2016	ODYSSEY HIGH FH	alirocumab	107	NR	NR	No	none	
Roth2016	ODYSSEY CHOICE I	alirocumab	803	NR	NR	No	BMI, smoking, DM, HT	
Stroes2016	ODYSSEY CHOICE II	alirocumab	228	NR	NR	No	smoking	
Teramoto 2016a	DFI12361	alirocumab	100	NR	NR	No	smoking	
Teramoto 2016b	ODYSSEY JAPAN	alirocumab	216	NR	NR	No	smoking, HT	
Teramoto 2017	ODYSSEY NIPPON	alirocumab	163	NR	NR	No	smoking, HT	
Leiter 2017	ODYSSEY DM - Insulin	alirocumab	517	NR	NR	No	LDL-c, HT	
Koh 2017	ODYSSEY KT	alirocumab	199	NR	NR	No	smoking, HT	
Blom 2020	ODYSSEY HoFH	alirocumab	69	NR	NR	No	smoking, DM, HT	
Giugliano 2012	LAPLACE-TIMI 57	evolocumab	631	CG	IVRS	No	BMI	
Raal 2012	RUTHERFORD-1	evolocumab	168	NR	IVRS	No	DM, HT	
Hirayama 2014	YUKAWA-1	evolocumab	310	NR	NR	No	BMI	
Blom 2014	DESCARTES	evolocumab	905	NR	IVRS	No	none	
Raal 2015	RUTHERFORD-2	evolocumab	331	CG	CVRS	No	DM, smoking, HT	
Kiyosue 2015	YUKAWA-2	evolocumab	404	NR	NR	No	BMI, HT	
Amgen 2016	FLOREY	evolocumab	45	NR	NR	No	BMI, smoking, DM, HT	
Nicholls2016	GLAGOV	evolocumab	968	NR	IVRS	No	none	
*Lipid lowering ezetimibe-controlled trials*								
Bays 2014	ODYSSEY OPTIONS I	alirocumab	355	NR	NR	No	smoking	
Roth 2014	ODYSSEY MONO	alirocumab	103	NR	NR	No	smoking, HT	
Cannon 2015	ODYSSEY COMBO II	alirocumab	720	NR	IVRS	No	smoking, HT	
Moriarty 2015	ODYSSEY ALTERNATIVE	alirocumab	251	NR	NR	No	none	
Farnier 2016	ODYSSEY OPTIONS II	alirocumab	204	NR	NR	No	smoking	
Han 2018	ODYSSEY EAST	alirocumab	615	NR	NR	No	smoking	
Stroes 2014	GAUSS-2	evolocumab	307	NR	NR	No	BMI	
Nissen 2016	GAUSS-3	evolocumab	218	CRP	IVRS/IWRS	No	none	
Koba 2020	GAUSS-4	evolocumab	61	NR	IVRS/IWRS	No	BMI	
*Lipid lowering placebo- and ezetimibe-controlled trials*								
Koren 2012	MENDEL-1	evolocumab	365	NR	IVRS	No	none	
Sullivan 2012	GAUSS-1	evolocumab	64	NR	IVRS	No	none	
Koren 2014	MENDEL-2	evolocumab	461	NR	NR	No	BMI	
Robinson 2014	LAPLACE-2	evolocumab	1678	NR	NR	No	BMI, smoking, HT	
*Clinical outcomes trials*								
Schwartz 2018	ODYSSEY OUTCOMES	alirocumab	18,924	NR	NR	No	none	
Sabatine 2017	FOURIER	evolocumab	27,564	CC	CC	No	none	

* only the groups included in our meta-analysis; HT = hypertension, BMI = Body Mass Index, DM = diabetes mellitus, NR = not reported, CG = computer generated, CRP = centralized randomisation process, CC = central computer, IVRS = interactive voice response system, CAS = central allocation system, CVRS = computerized voice response system, IWRS = interactive web response system

**Table 2:** Range and direction of baseline differences in evolocumab and alirocumab trials

	PCSK9 inhibitor versus placebo (*n* = 34)			PCSK9 inhibitor versus ezetimibe (*n* = 13)			PCSK9 inhibitor versus placebo or ezetimibe (*n* = 43)		
Patient characteristic	Difference, range^#^	Trials, *n*-/n0/*n*+^$^	Sign test, *p* ^&^	Difference, range^#^	Trials, *n*-/n0/*n*+^$^	Sign test, *p* ^&^	Difference, range^#^	Trials, *n*-/n0/*n*+^$^	Sign test, *p* ^&^
Age in years, mean	-3.1 to 4.1	15/5/10	0.212	-1.9 to 4.0	5/0/8	0.291	-3.1 to 4.1	20/5/18	0.436
Male gender, %	-19.6 to 25.8	20/1/9	0.031	-11.4 to 7.2	4/0/9	0.133	-19.6 to 25.8	24/1/18	0.220
LDL-c in mg/dl, mean	-7.7 to 35.4	10/2/18	0.093	-4.0 to 13.4	6/0/7	0.500	-7.7 to 35.4	16/2/25	0.106
Body mass index, mean	-1.5 to 1.5	11/1/10	0.500	-0.7 to 1.7	4/0/5	0.500	-1.5 to 1.7	15/1/15	0.572
Diabetes mellitus, %	-12.6 to 13.2	11/1/12	0.500	-9.4 to 17.4	7/1/5	0.387	-12.6 to 17.4	18/2/17	0.500
Smoking, %	-12.6 to 7.6	10/0/8	0.407	-6.8 to 4.7	3/0/4	0.500	-12.6 to 7.6	13/0/12	0.500
Hypertension, %	-24.0 to 13.7	9/0/8	0.500	-11.5 to 10.0	4/0/6	0.377	-24.0 to 13.7	13/0/14	0.500

LDL-c stands for LDL-cholesterol # the baseline difference for each trial was calculated as the mean or percentage in the PCSK9 inhibitor group minus the mean or percentage of the control group $ n- for the number of trials with a negative baseline imbalance (f.i. lower age in PCSK9 inhibitor versus placebo group), and n0 for the number of trials with no baseline imbalance (f.i. similar mean age in PCSK9 inhibitor and placebo group), and n + stands for the number of trials with a positive baseline imbalance (f.i. higher age in PCSK9 inhibitor versus placebo group) & one-sided sign-test to test whether the proportion of studies that reported an imbalance in the most common direction could be attributed to chance

### Pooled baseline differences and heterogeneity

Table 3 shows the pooled baseline differences between the drug and control groups. Pooled baseline differences were mostly small. The pooled difference in BMI was statistically significant in the placebo comparisons (-0.16; 95% CI -0.24 to -0.09) and all comparisons (-0.15; 95% CI -0.22 to -0.07). The baseline difference in BMI in the FOURIER trial drove the statistical significance of these differences, but the pooled difference was present in the smaller placebo-controlled lipid lowering trials as well (-0.18; 95% CI -0.42 to 0.06). The difference in proportion of participants with hypertension was statistically significant for all comparisons (0.01; 95% CI 0.00 to 0.02). Again, the pooled difference in proportion of participants with hypertension was statistically significant due to the baseline imbalance in the ODYSSEY OUTCOMES trial (35% of weight), but the smaller lipid-lowering trials showed a similar imbalance (0.01; 95% CI -0.02 to 0.03).

Table 3 also shows the heterogeneity in the baseline imbalances. In the placebo comparisons, six of seven pooled baseline differences had a heterogeneity > 0% when there should be none. The heterogeneity was statistically significant for the difference in proportion of males (I^2^ 41%; 95% CI 9–62; *p* = 0.011). In the ezetimibe comparisons, five of seven baseline differences showed heterogeneity > 0%. The heterogeneity was statistically significant for difference in the proportion with DM (I^2^ 50%; 95% CI 5–74; *p* = 0.020). For the combined comparisons, five of seven pooled baseline differences showed heterogeneity. This heterogeneity was statistically significant for imbalances in the proportion of males (I^2^ 31%; 95% CI 0–53; *p* = 0.029, mean BMI (I^2^ 33%; 95% CI 0–57; *p* = 0.039), proportion of participants with DM (I^2^ 32%; 95% CI 0–55; *p* = 0.035) and proportion of participants with hypertension (I^2^ 36%; 95% CI 0–60; *p* = 0.033).

### Imbalances in SDs

All SDs around means of age, LDL-c were reported, but the means of BMI and corresponding SDs were missing for 12 of 43 comparisons. Table 4 shows the range in imbalances in SDs. In some studies, the SDs differed between the drug and control groups: the differences for SDs of age varied between − 1.7 and 3.0 years in the placebo comparisons and − 2.1 to 2.7 years in ezetimibe comparisons. For the differences in SDs of LDL-c the ranges were − 21.2 to 23.7 mg/dl respectively − 15.7 to 15.8 mg/dl, and for differences in SDs of BMI − 0.8 to 1.1 kg/m^2^ respectively − 1.5 to 1.7 kg/m^2^.

Table 4 also shows that statistically significantly more often drug groups had a larger SD than the placebo group around mean age (21 versus 8; *p* = 0.012) and mean LDL-c (20 versus 8; *p* = 0.018). For ezetimibe comparisons, numerically but not statistically significantly more drug groups had a larger SD for all three variables. All comparisons together showed that the drug groups had a larger SD than the control group more often than expected for age (28 versus 13; *p* = 0.01), LDL-c (28 versus 13; *p* = 0.014) and BMI (20 versus 10; *p* = 0.049).

Table 5 presents the pooled SDs of age, LDL-c and BMI for the drug and control groups. The pooled SD of the drug groups was larger than the pooled SD of the control groups in eight of nine analyses, and smaller in one analysis. None of the differences was statistically significant. The 28 smaller lipid-lowering trials and 2 clinical outcomes trials showed similar differences in SDs between drug and control group, with one exception that the SDs of LDL-c in the groups of the clinical outcomes trials were the same (supplemental Table 5).

**Fig. 2:**
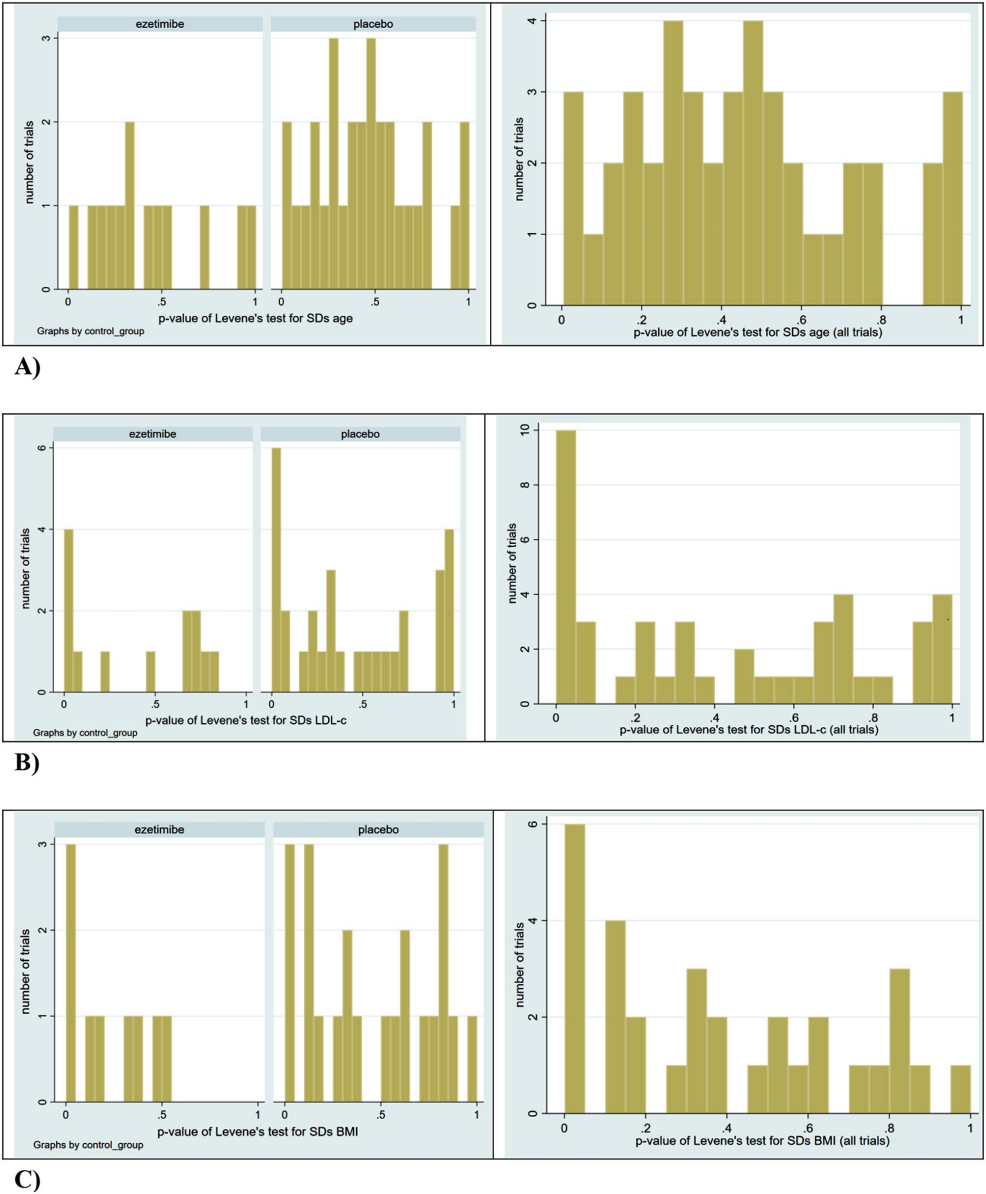
Histogram of p-values of Levene’s test for differences in SDs around (**A**) mean age, (**B**) mean LDL-c, and (**C**) mean BMI; left panel represents p-values by control group (ezetimibe or placebo); right panel represents p-values for all comparisons

Figure 2 shows the distribution of p-values generated by Levene’s test for the difference between SDs of the study groups per trial. The p-values for the SDs of age seemed fairly evenly distributed between 0 and 1. The distributions for SDs of LDL-c and BMI were skewed to the left: the p-value was below 0.05 in 10 of 43 comparisons (23%) for the former, and in 6 of 31 comparisons (19%) for the latter.

### Alirocumab and evolocumab separately

The alirocumab and evolocumab trials showed similar patterns for baseline differences and heterogeneity as the main results (supplemental Table 1). Nevertheless, in the alirocumab trials, heterogeneity was statistically significantly high for the baseline difference in the proportion of males (42%; 95% CI 4–65; *p* = 0.02) and the proportion of DM (I^2^ 45%; 95% CI 6–67; *p* = 0.02) (supplemental Table 2). The alirocumab and evolocumab trials also showed similar patterns in the directions of differences in SDs and in the pooled SDs as seen in the main results (supplemental Tables 3 and supplemental Table 4).

**Table 3:** Pooled baseline differences with heterogeneity in evolocumab and alirocumab trials

	PCSK9 inhibitor versus placebo (*n* = 30)		PCSK9 inhibitor versus ezetimibe (*n* = 13)		All PCSK9 inhibitor versus placebo or ezetimibe (*n* = 43)	
Patient characteristic	Pooled difference^, MD or RD (95%CI)	Heterogeneity *p*^$^; I^2^ (95% CI)	Pooled difference^, MD or RD (95%CI)	Heterogeneity, *p*^$^; I^2^ (95% CI)	Pooled difference^, MD or RD (95%CI)	Heterogeneity, *p*^$^; I^2^ (95% CI)
Age in years, mean	-0.04 (-0.19 to 0.12)	0.748; 0 (0–41)	0.01 (-0.61 to 0.62)	0.347; 10 (0–48)	-0.03 (-0.18 to 0.12)	0.692; 0 (0–35)
Male gender, proportion	-0.00 (-0.01 to 0.01)	0.011; 41 (9–62)	0.03 (-0.00 to 0.06)	0.756; 0 (0–57)	0.00 (-0.01 to 0.01)	0.029; 31 (0–53)
LDL-c in mg/dl, mean	0.02 (-0.39 to 0.44)	0.405; 4 (0–33)	1.37 (-0.95 to 3.69)	0.593; 0 (0–57)	0.07 (-0.34 to 0.47)	0.484; 0 (0–35)
Body mass index, mean	-0.16 (-0.24 to -0.09)*	0.051; 36 (0–62)	0.28 (-0.10 to 0.13)	0.346; 11 (0–69)	-0.15 (-0.22 to -0.07)*	0.027; 36 (0–58)
DM, proportion	0.00 (-0.01 to 0.01)	0.116; 27 (0–56)	0.01(-0.02 to 0.03)	0.020; 50 (5–74)	0.00 (-0.01 to 0.01)	0.035; 32 (0–55)
Smoking, proportion	-0.00 (-0.01 to 0.01)	0.342; 9 (0–45)	-0.02 (-0.05 to 0.02)	0.212; 28 (0–69)	-0.00 (-0.01 to 0.00)	0.317; 10 (0–43)
Hypertension, proportion	0.01 (-0.00 to 0.02)	0.096; 33 (0–62)	0.02 (-0.02 to 0.05)	0.052; 46 (0–74)	0.01 (0.00 to 0.02)#	0.033; 36 (0–60)

LDL-c stand for LDL cholesterol and DM for diabetes mellitus; ^ drug versus control group; $ Chi2; * *p* < 0.01; # *p* < 0.05

**Table 4:** Range and direction of differences between standard deviations around baseline means in evolocumab and alirocumab trials

	PCSK9 inhibitor versus placebo (*n* = 30)			PCSK9 inhibitor versus ezetimibe (*n* = 13)			PCSK9 inhibitor versus placebo or ezetimibe (*n* = 43)		
Patient characteristic	Difference, range^#^	Trials, *n*-/n0/*n*+^$^	Sign test, *p* ^&^	Difference, range^#^	Trials, *n*-/n0/*n*+^$^	Sign test, *p* ^&^	Difference, range^#^	Trials, *n*-/n0/*n*+^$^	Sign test, *p* ^&^
Age in years, SD of mean	-1.7 to 3.0	8/1/21	0.012	-2.1 to 2.7	5/1/7	0.387	-2.1 to 3.0	13/2/28	0.014
LDL-c in mg/dl, SD of mean	-21.2 to 23.7	8/2/20	0.018	-15.7 to 15.8	5/0/8	0.291	-21.2 to 23.7	13/2/28	0.014
BMI, SD of mean	-0.8 to 1.1	8/1/13	0.192	-1.5 to 1.7	2/0/7	0.090	-1.5 to 1.7	10/1/20	0.049

BMI stands for body mass index, LDL-C for LDL-cholesterol # the difference for each trial was calculated as the SD in the PCSK9 inhibitor group minus the SD of the control group $ n- for the number of trials with a negative difference (f.i. lower SD in PCSK9 inhibitor versus placebo group), and n0 for the number of trials with no difference (f.i. similar SD in PCSK9 inhibitor versus placebo group), and n + stands for the number of trials with a positive difference (f.i. higher SD in PCSK9 inhibitor versus placebo group); if n-, n0 and n + do not add up to the total n of trials with this comparison mentioned in the column heading, the SDs are missing for the rest of the trials. & one-sided sign-test to test whether the proportion of studies that reported an imbalance in the most common direction could be attributed to chance

**Table 5:** Pooled standard deviations around baseline means in evolocumab and alirocumab trials

	PCSK9 inhibitor versus placebo (*n* = 30)		PCSK9 inhibitor versus ezetimibe (*n* = 13)		PCSK9 inhibitor versus placebo or ezetimibe (*n* = 43)	
Patient characteristic	Pooled SD, drug vs. control	*p*^	Pooled SD, drug vs. control	*p*^	Pooled SD, drug vs. control	*p*^
Age in years, SD of mean	9.58 vs. 9.29	0.338	10.51 vs. 10.03	0.403	9.88 vs. 9.54	0.290
LDL-c in mg/dl, SD of mean	29.75 vs. 27.84	0.675	44.55 vs. 44.94	0.950	34.30 vs. 33.15	0.806
BMI, SD of mean	4.78 vs. 4.75	0.308	5.70 vs. 5.18	0.515	5.06 vs. 4.83	0.117

BMI stands for body mass index, LDL-c for LDL-cholesterol ^ t-test for means (in this case averages of SDs)

**Table 6:** Relationship of baseline imbalances with clinical outcomes in evolocumab and alirocumab trials

	LDL-c		MACE		Serious adverse event		Any adverse event		Mortality	
Imbalance, drug vs. control group	*N*	Effect on absolute reduction (95% CI)	*N*	Effect on OR (95% CI)	*N*	Effect on OR (95% CI)	*N*	Effect on OR (95% CI)	*N*	Effect on OR (95% CI)
Age, per year older	39	-1.34 (-0.16 to 0.23)	40	-0.04 (-0.35 to 0.28)	41	-0.01 (-0.17 to 0.15)	40	0.07 (-0.01 to 0.14)	43	0.16 (-0.25 to 0.58)
Males, per 1% more	39	0.02 (-0.01 to 0.06)	40	-0.01 (-0.04 to 0.03)	41	0.02 (-0.01 to 0.04)	40	-0.01 (-0.02 to 0.01)	43	-0.00 (-0.07 to 0.06)
LDL-c, per 1 mg/dl more	39	-0.02 (-0.06 to 0.02)	40	0.00 (-0.07 to 0.07)	41	-0.02 (-0.06 to 0.02)	40	-0.01 (-0.03 to 0.01)	43	-0.01 (-0.10 to 0.07)
BMI, per 1 point more	28	0.20 (-0.27 to 0.67)	29	-0.03 (-0.37 to 0.32)	29	0.02 (-0.28 to 0.31)	28	-0.08 (-0.25 to 0.08)	31	-0.56 (-1.10 to -0.02)^*^
DM, per 1% more	35	-0.00 (-0.05 to 0.06)	36	0.00 (-0.05 to 0.06)	35	-0.02 (-0.05 to 0.02)	34	0.02 (0.01 to 0.04)^*^	37	-0.05 (-0.14 to 0.04)
Smokers, per 1% more	22	0.01 (-0.03 to 0.06)	24	-0.03 (-0.11 to 0.06)	23	-0.01 (-0.04 to 0.06)	23	-0.01 (-0.04 to 0.02)	25	-0.09 (-0.24 to 0.07)
Hypertension, per 1% more	25	-0.00 (-0.04 to 0.04)	27	-0.01 (-0.05 to 0.04)	25	0.02 (-0.02 to 0.05)	25	-0.00 (-0.02 to 0.02)	27	-0.05 (-0.13 to 0.04)

LDL-c stand for LDL-cholesterol, BMI for body mass index and DM for diabetes mellitus; N refers to number of studies included in the meta-regression analysis; * *p* < 0.05

### Association with clinical outcomes

Table 6 presents the association between baseline differences and clinical outcomes. Most baseline differences did not show a statistically significant association with the risk of the clinical outcomes, but various associations seemed strong. E.g., a one-year higher mean age in the drug than control group was associated with a 16% higher relative risk of all-cause mortality (beta 0.16; 95% CI -0.25 to 0.58). A one-point higher mean BMI in the drug versus control group was associated with an 8% lower relative risk of any adverse events (beta − 0.08; 95% CI -0.25 to 0.08), and a 56% lower relative risk of all-cause mortality (beta − 0.56; 95% CI -1.10 to -0.02). In addition, 1% more diabetic patients in the drug versus control group was statistically significantly associated with a 2% higher relative risk of any adverse events (beta 0.02; 95% CI 0.01 to 0.04).

When excluding the two clinical outcomes trials from the meta-regression analysis, the effect of a higher BMI in the intervention versus control group became smaller and was not statistically significant anymore (supplemental Table 6). The other results did not change substantially.

## Discussion

In our study of 43 randomized trials of evolocumab or alirocumab, many trials showed baseline imbalances, but they were not adjusted for. Pooled baseline imbalances were small but showed high heterogeneity for BMI, DM, smoking and hypertension, when none is expected. In addition, statistically significantly more trials showed PCSK9 inhibitor groups with larger SDs around age, BMI and LDL-c than the control groups. A higher mean BMI in the PCSK9 inhibitor versus control group was associated with a decreased risk of all-cause mortality, and a higher proportion of diabetic patients with an increased risk of any adverse event.

Our study showed that randomisation has not produced comparable groups in alirocumab and evolocumab trials. Some individual trials showed clear clinically relevant imbalances, and in many trials, the drug groups had higher SDs for age, BMI and LCL-c than the control group. The discrepancy in SDs is related to an uneven distribution of participants over the groups, even if the means are (almost) similar. Box 1 illustrates that a somewhat lower mean age and a higher SD in the drug versus control group reflects more young people in the former than is the latter. If the investigated drug is effective in young people and not in older people, this distribution favours the drug group.

**Figure:**
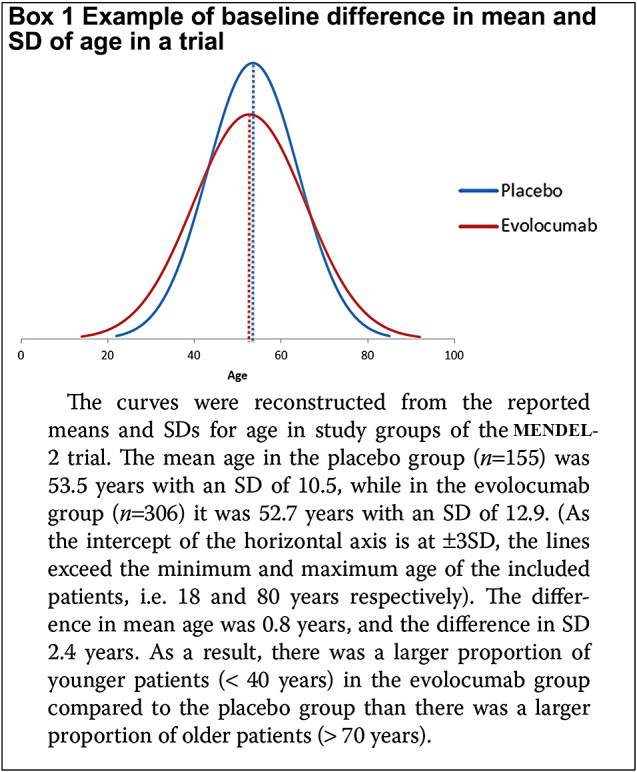

### Incomparable groups

To our knowledge, only a few other meta-epidemiological studies have also assessed pooled baseline imbalances and heterogeneity in randomized trials. One study focused on imbalances in age and blood pressure in 391 trials about exercise and antihypertensive medication [67]. There was a statistically significant pooled difference in age favouring the intervention over the control groups. Also, there was heterogeneity for 16 of 29 comparisons. Another study investigated baseline imbalances in age in 503 trials included in 12 published meta-analyses [68]. Two meta-analyses showed a significantly higher mean age in the intervention than the control group, and five heterogeneity in age differences. Yet another study reported small pooled baseline imbalances, but heterogeneity in 4 of 6 investigated imbalances in 23 trials about antipsychotics in dementia [15]. We could not identify other meta-epidemiological studies that examined baseline differences in SDs.

Our study also showed that baseline imbalances may affect the validity of the treatment effect of PCSK9 inhibitors reported in systematic reviews [69–71]. In particular, imbalances in BMI and proportion of diabetic patients seemed to have influenced effects on safety outcomes. One or both factors showed imbalances in the clinical outcomes trials, which had very high weights in the meta-analyses [69–71]. These factors should have been adjusted for in the original trials [14]. Moreover, as obesity and diabetes are related, they will not have been independently distributed in the trial populations.

### Implications

The heterogeneity in baseline imbalances and differences in SDs raise concerns about the adequacy of randomization methods in the included alirocumab and evalocumab trials. In the context of randomization, one does not expect heterogeneity, because the goal of randomization is to not get baseline differences between groups (‘no effect’), let alone differences in those differences. SDs may differ between study groups by chance, but Levene’s test showed that more than 1 in 20 trials had a statistically significant difference in SDs of BMI and LDL-c. This is especially disconcerting because 34 of the 43 trials had more participants in the drug versus control group, often twice as many, and SDs decrease with an increasing number of persons. Only 30% of trials reported the use of a central computerized systems for randomisation, which is currently considered the gold standard. The lack of information about the randomization procedures in the majority of trials hinders a detailed assessment of the quality of the randomisation design and its relation to our findings.

Our findings suggest the need for objective assessment of risk of bias as part of systematic reviews. Published systematic reviews about PCSK9 inhibitors trials used conventional risk of bias tools [69–71], and the risk of bias in the randomization domain was considered low in most or all trials. In our study, incomplete or missing information on random sequence generation and allocation concealment in the included trials implies an uncertain risk of bias in the randomization method, leading to an overall uncertain risk of bias.

For objective assessment, imbalances in prognostic factors need to be examined, including age, which is a potent predictor of clinical outcomes in many trials and seldom not reported [2]. Another option is to study baseline p-value distributions in all reported continuous and categorical variables [72]. In addition, methods to adjust for baseline imbalances in meta-analyses have been proposed [13, 73]. To enable the evaluation of the quality of randomization procedures and its association with baseline imbalances, authors need to describe the procedures and all prognostic baseline characteristics per study group according to the CONSORT guidelines for reporting randomized trials.

### Strengths and limitations

A strength of this study is that we used an objective method to identify risk of bias due baseline imbalances. This approach may be a valuable addition to standard assessments with a risk of bias tool, which often yield discrepant results [74, 75].

A limitation of our study is that our analyses depended on the availability of baseline data in the publications. This was often lacking for BMI, DM and smoking. Another limitation of our study was the number of included trials. If many more studies had been available, a multivariate meta-regression analysis could have performed to study whether baseline imbalances were related to each other. A final limitation is that we did not correct for multiple-testing. Although some authors have made suggestions to overcome multiple testing in standard reviews [76], no formal guideline for correction of multiple testing in meta-epidemiological studies has been proposed. Consequently, we may have identified statistically significant results by chance, especially in the meta-regression analyses. Nevertheless, the consistency in the statistically significant differences observed for most standard deviations cannot be explained by multiple testing.

## Conclusion

Clinically relevant baseline imbalances in evolocumab and alirocumab trials were not adjusted for and may have biased the measured effects on key clinical outcomes. In addition, heterogeneity in baseline imbalances and systematic differences in SDs between study groups in PCSK9 inhibitor trials raise concerns about the effectiveness of the randomization procedures. Baseline characteristics of the study groups can be used to assess risk of bias in trials objectively as part of systematic reviews.

### Electronic supplementary material

Below is the link to the electronic supplementary material.

Supplementary Material 1

## Data Availability

All data from the included studies are publicly available.
