# Supplementary material for: Quantitative assessment of baseline imbalances in evolocumab and alirocumab trials: a meta-epidemiological study
Source: BMC Med Res Methodol. 2024 Jun 22;24:137. doi: 10.1186/s12874-024-02260-z (PMC11193208; doi:10.1186/s12874-024-02260-z)
Supplement: Supplementary file 1 — Supplementary Material 1 [file 12874_2024_2260_MOESM1_ESM.docx]

**Supplemental tables**

**Supplemental table S1 Range and direction of baseline differences in alirocumab and evolocumab trials separately**

|  | Alirocumab trials (n=23),  drug versus control group | | | Evolocumab trials (n=20),  drug versus control group | | |
| --- | --- | --- | --- | --- | --- | --- |
| Patient characteristic | **Difference, range^#^** | **Trials,**  **n-/n0/n+^$^** | **Sign test,**  **p^&^** | **Difference, range^#^** | **Trials,**  **n-/n0/n+^$^** | **Sign test,**  **p^&^** |
| Age in years, mean | -3.1 to 4.1 | 11/3/9 | 0.412 | -1.4 to 4.0 | 9/2/9 | 0.593 |
| Male gender, % | -14.7 to 25.8 | 14/0/9 | 0.202 | -19.6 to 16.1 | 10/1/9 | 0.500 |
| LDL-c in mg/dl, mean | -5.8 to 35.4 | 8/1/14 | 0.143 | -7.7 to 13.4 | 8/1/11 | 0.324 |
| Body mass index, mean | -1.5 to 1.7 | 10/1/11 | 0.500 | -0.8 to 1.7 | 5/0/4 | 0.500 |
| Diabetes mellitus, % | -12.6 to 17.4 | 7/1/12 | 0.180 | -9.4 to 7.6 | 11/1/5 | 0.105 |
| Smoking, % | -12.6 to 5.7 | 4/0/5 | 0.500 | -8.7 to 7.6 | 9/0/7 | 0.402 |
| Hypertension, % | -24.0 to 11.2 | 5/0/7 | 0.387 | -11.5 to 13.7 | 8/0/7 | 0.500 |

LDL-c stands for LDL-cholesterol * p<0.05 # the baseline difference for each trial was calculated as the mean or percentage in the PCSK9 inhibitor group minus the mean or percentage of the control group $ n- for the number of trials with a negative difference (f.i. lower SD in PCSK9 inhibitor versus placebo group), and n0 for the number of trials with no difference (f.i. similar SD in PCSK9 inhibitor versus placebo group), and n+ stands for the number of trials with a positive difference (f.i. higher SD in PCSK9 inhibitor versus placebo group); if n-, n0 and n+ do not add up to the total n of trials with this comparison mentioned in the column heading, the SDs are missing for the rest of the trials. & one-sided sign-test to test whether the proportion of studies that reported an imbalance in the most common direction could be attributed to chance

**Supplemental Table S2 Pooled baseline differences with heterogeneity for evolocumab and alirocumab trials separately**

|  | Alirocumab trials (n=23),  intervention versus control group | | Evolocumab trials (n=20),  intervention versus control group | |
| --- | --- | --- | --- | --- |
| Patient characteristic | **Pooled difference,**  **MD or RD (95%CI)** | **Heterogeneity,**  **p^; I^2^ (95% CI)** | **Pooled difference,**  **MD or RD (95%CI)** | **Heterogeneity,**  **p^; I^2^ (95% CI)** |
| Age in years, mean | -0.07 (-0.30 to 0.16) | 0.617; 0 (0-45) | -0.01 (-0.18 to 0.12) | 0.567; 0 (0-48) |
| Male gender, proportion | 0.00 (-0.01 to 0.01) | 0.020; 42 (4-65) | 0.00 (-0.01 to 0.01) | 0.221; 19 (0-53) |
| LDL-c in mg/dl, mean | 0.32 (-0.47 to 1.12) | 0.740; 0 (0-45) | -0.03 (-0.50 to 0.45) | 0.206; 20 (0-53) |
| Body mass index, mean | 0.02 (-0.09 to 0.13) | 0.316; 11 (0-45) | -0.29 (-0.39 to -0.18)* | 0.442; 0 (0-65) |
| Diabetes mellitus, proportion | 0.00 (-0.01 to 0.01) | 0.017; 45 (6-67) | 0.00 (-0.01 to 0.01) | 0.292; 14 (0-51) |
| Smoking, proportion | 0.00 (-0.01 to 0.01) | 0.158; 33 (0-69) | -0.01 (-0.02 to 0.00) | 0.530; 0 (0-52) |
| Hypertension, proportion | 0.02 (0.01 to 0.03)^#^ | 0.282; 16 (0-56) | 0.00 (-0.01 to 0.01) | 0.063; 39 (0-67) |

LDL-c stands for LDL-cholesterol * p<0.01; # p<0.05; ^ Chi2

**Supplemental table S3 Range and direction of differences between SDs for alirocumab and evolocumab trials separately**

|  | Alirocumab trials (n=23),  intervention versus control group | | | Evolocumab trials (n=20)  intervention versus control group | | |
| --- | --- | --- | --- | --- | --- | --- |
| Patient characteristic | **Difference, range^#^** | **Trials,**  **n-/n0/n+^$^** | **Sign test,**  **p^&^** | **Difference, range^#^** | **Trials,**  **n-/n0/n+^$^** | **Sign test,**  **p^&^** |
| Age in years, SD of mean | -1.7 to 3 | 8/1/14 | 0.143 | -2.1 to 2.7 | 5/1/14 | 0.032 |
| LDL-c in mg/dl, SD of mean | -21.1 to 14.8 | 7/1/15 | 0.067 | -4.3 to 23.7 | 6/1/13 | 0.084 |
| BMI, SD of mean | -0.8 to 1.7 | 8/0/14 | 0.143 | -1.5 to 1.3 | 2/1/6 | 0.145 |

LDL-c stands for LDL-cholesterol, BMI for body mass index * p<0.05

**Supplemental Table 4 Pooled standard deviations, and Levene’s p-value for evolocumab and alirocumab separately**

|  | Alirocumab trials (n=23) | | Evolocumab trials (n=20) | |
| --- | --- | --- | --- | --- |
| Patient characteristic | **Pooled SD,**  **drug vs control; p^** | **Levene’s test*, n <.05/>=05/missing SDs** | **Pooled SD,**  **drug vs control; p^** | **Levene’s test*, n <.05/>=05/missing SDs** |
| Age in years, SD of mean | 10.53 vs 10.12; 0.68 | 0/ 23/ 0 | 10.51 vs 10.01; 0.22 | 3/ 17/ 0 |
| LDL-c in mg/dl, SD of mean | 36.26 vs 34.36; 0.47 | 4/ 18/ 1 | 26.70 vs 24.21; 0.48 | 6/ 14/ 0 |
| BMI, SD of mean | 4.81 vs 4.69; 0.58 | 3/ 15/ 5 | 4.86 vs 4.65; 0.32 | 2/ 7/11 |

LDL-c stands for LDL-cholesterol, BMI for body mass index; ^ t-test for means; * Levene’s test for the difference in SD between study groups of each trial

**Supplemental Table 5 Pooled standard deviations in lipid-lowering and clinical outcomes trials**

|  | PCSK9 inhibitor versus placebo group,  pooled SD*; p^; n | |
| --- | --- | --- |
| Patient characteristic | **Lipid-lowering efficacy trials** | **Clinical outcomes trials** |
| Age in years, SD of mean | 10.62 vs 10.21; 0.215; 28 | 9.18 vs 9.11; 0.723; 2 |
| LDL-c in mg/dl, SD of mean | 38.88 vs 37.10; 0.595; 28 | 25.79 vs 25.79; 0.999; 2 |
| BMI, SD of mean | 5.48 vs 5.36; 0.654; 20 | 4.60 vs 4.55; 0.365; 2 |

LDL-c stands for LDL-cholesterol, BMI for body mass index; * drug group vs placebo group; ^ t-test for means

**Supplemental table 6 Relationship of baseline imbalances with effects on outcomes: sensitivity analysis without clinical outcomes trials**

|  | LDL-c | | MACE | | Serious adverse event | | Any adverse event | | Mortality | |
| --- | --- | --- | --- | --- | --- | --- | --- | --- | --- | --- |
| Imbalance,  drug vs control group | **N** | **Effect on absolute**  **reduction (95% CI)** | **N** | **Effect on OR**  **(95% CI)** | **N** | **Effect on OR**  **(95% CI)** | **N** | **Effect on OR**  **(95% CI)** | **N** | **Effect on OR**  **(95% CI)** |
| Age, per year older | 39 | -0.03 (-0.16 to 0.23) | 38 | -0.05 (-0.37 to 0.28) | 41 | -0.01 (-0.17 to 0.15) | 38 | 0.06 (-0.01 to 0.14) | 41 | 0.16 (-0.26 to 0.58) |
| Males, per 1% more | 39 | 0.02 (-0.01 to 0.06) | 38 | -0.01 (-0.05 to 0.03) | 41 | 0.02 (-0.01 to 0.04) | 38 | -0.01 (-0.02 to 0.01) | 41 | -0.00 (-0.07 to 0.07) |
| LDL-c, per 1 mg/dl more | 39 | -0.02 (-0.06 to 0.02) | 38 | 0.00 (-0.07 to 0.07) | 41 | -0.02 (-0.06 to 0.02) | 38 | -0.01 (-0.03 to 0.01) | 41 | -0.00 (-0.09 to 0.08) |
| BMI, per 1 point more | 28 | 0.20 (-0.27 to 0.67) | 27 | -0.09 (-0.74 to 0.56) | 29 | 0.02 (-0.28 to 0.31) | 26 | -0.10 (-0.27 to 0.08) | 29 | -0.21 (-1.16 to 0.73) |
| DM, per 1% more | 35 | -0.00 (-0.05 to 0.06) | 34 | 0.00 (-0.06 to 0.06) | 35 | -0.02 (-0.05 to 0.02) | 32 | 0.02 (0.06 to 0.04)^*^ | 35 | -0.04 (-0.14 to 0.05) |
| Smokers, per 1% more | 22 | 0.01 (-0.03 to 0.06) | 22 | -0.05 (-0.15 to 0.05) | 23 | -0.01 (-0.04 to 0.06) | 21 | -0.01 (-0.04 to 0.02) | 23 | -0.07 (-0.23 to 0.09) |
| Hypertension, per 1% more | 25 | -0.00 (-0.04 to 0.04) | 25 | -0.01 (-0.07 to 0.06) | 25 | 0.02 (-0.02 to 0.05) | 23 | -0.00 (-0.02 to 0.02) | 25 | -0.02 (-0.11 to 0.07) |

LDL-c stand for LDL-cholesterol, BMI for body mass index and DM for diabetes mellitus; N refers to number of studies included in the meta-regression analysis; * p<0.05
